# Mediterranean Diet and Olive Oil Redox Interactions on Lactate Dehydrogenase Mediated by Gut *Oscillibacter* in Patients with Long-COVID-19 Syndrome

**DOI:** 10.3390/antiox13111358

**Published:** 2024-11-06

**Authors:** Amanda Cuevas-Sierra, Victor de la O, Andrea Higuera-Gómez, Lourdes Chero-Sandoval, Begoña de Cuevillas, María Martínez-Urbistondo, Victor Moreno-Torres, Ilduara Pintos-Pascual, Raquel Castejón, J. Alfredo Martínez

**Affiliations:** 1 Precision Nutrition and Cardiometabolic Health, IMDEA-Food Institute (Madrid Institute for Advanced Studies), Campus of International Excellence (CEI) UAM+CSIC, 28049 Madrid, Spain; victor.delao@alimentacion.imdea.org (V.d.l.O.); andrea.higuera@alimentacion.imdea.org (A.H.-G.); lourdeschero88@gmail.com (L.C.-S.); begona.decuevillas@alimentacion.imdea.org (B.d.C.); jalfredo.martinez@imdea.org (J.A.M.); 2 Faculty of Health Sciences, International University of La Rioja (UNIR), 26006 Logroño, Spain; victor.moreno.torres.1988@gmail.com; 3 Department of Endocrinology and Nutrition, University Clinical Hospital, University of Valladolid, 47002 Valladolid, Spain; 4 Internal Medicine Service, Puerta de Hierro Majadahonda University Hospital, 28222 Madrid, Spain; mmurbistondo@gmail.com (M.M.-U.); ilduarapintos@gmail.com (I.P.-P.); 5 Centro de Investigación Biomédica en Red de la Fisiopatología de la Obesidad y Nutrición (CIBERobn), Instituto de Salud Carlos III, 28029 Madrid, Spain; 6 Centro de Medicina y Endocrinología, Universidad de Valladolid, 47005 Valladolid, Spain

**Keywords:** Mediterranean diet, extra virgin olive oil, *Oscillibacter*, lactate dehydrogenase, health benefits, antioxidant

## Abstract

Chronic viral inflammation is associated with oxidative stress and changes in gut microbiota. The Mediterranean diet (MD), with recognized anti-inflammatory and antioxidant properties, modulates gut microorganisms, specifically on the interaction between extra virgin olive oil, a key component of the MD with well-documented antioxidant effects. This study investigated the influence of adherence to MD and antioxidant-rich foods (extra virgin olive oil) on biochemical, inflammatory, and microbiota profiles in patients with chronic inflammation defined as a prolonged inflammatory response due to immune dysregulation following the acute phase of the viral infection. Participants were classified into low (n = 54) and high (n = 134) MD adherence groups (cut-off of 7 points based on previous studies utilizing the same threshold in the assessment of MD adherence). Gut microbiota was sequenced using the 16S technique, and the adherence to MD was assessed using a validated questionnaire for a Spanish population. High adherence to the MD was linked to significant improvements in inflammatory and oxidative stress markers, including reductions in LDL-cholesterol, glucose, and lactate dehydrogenase (LDH) levels, an indicative of redox balance, as well as a significant higher consumption of antioxidant foods. Moreover, gut microbiota analysis revealed distinct compositional shifts and a lower abundance of the *Oscillibacter* genus in the high adherence group. Notably, a significant interaction was observed between MD adherence and extra virgin olive oil consumption, with *Oscillibacter* abundance influencing LDH levels, suggesting that the MD antioxidant properties may modulate inflammation through gut microbiota-mediated mechanisms. These findings provide new evidence that adherence to the Mediterranean diet can reduce inflammatory markers in patients with long-COVID-19, a population that has not been extensively studied, while also highlighting the potential role of the bacterial genus *Oscillibacter* in modulating this effect.

## 1. Introduction

Chronic inflammation represents a complex and persistent pathophysiological state that significantly impacts health and well-being, which is related to oxidative stress and immunity [1]. Thus, persistent inflammation induced by trauma, metabolic, or viral injuries is characterized by a prolonged and dysregulated immune response, leading to tissue damage, and contributing to the development of some chronic diseases [1]. In chronic inflammatory states, redox imbalance plays a crucial role by exacerbating oxidative stress through the excessive production of reactive oxygen species (ROS) and reactive nitrogen species (RNS), which overwhelm the body’s antioxidant defenses [2]. This imbalance not only damages cellular components such as lipids, proteins, and DNA but also amplifies pro-inflammatory signaling pathways, creating a vicious cycle that sustains inflammation [3]. Among the diverse manifestations of chronic inflammation, long-hauler patients—individuals experience a prolonged inflammatory response due to immune dysregulation following the acute phase of the viral infection—pose a significant clinical challenge [4]. These patients frequently suffer from a range of debilitating symptoms, including fatigue, dyspnea, cognitive impairment, and musculoskeletal pain, all of which severely affect the quality of life and functional capacity, as well as the immune and inflammatory mechanisms [5]. In this context, lactate dehydrogenase (LDH), a widely used biomarker of tissue damage, inflammation, and oxidative stress, reflects cellular metabolic activity, being implicated in disease severity and prognosis [2,6].

Additionally, emerging research has highlighted that long-hauler infected patients often present alterations in gut microbiota composition, suggesting a link between chronic inflammation and microbial dysbiosis [7]. In chronic inflammatory conditions, such as inflammatory bowel disease, metabolic syndrome, or long-COVID-19, dysbiosis contributes to an impaired gut barrier function, often referred to as “leaky gut”. This increased intestinal permeability allows bacterial endotoxins to translocate into the systemic circulation, triggering systemic immune activation [8]. This microbial translocation exacerbates inflammation through the activation of pattern recognition receptors. Thus, chronic inflammation status can perpetuate dysbiosis by creating a hostile gut environment—marked by oxidative stress, immune dysregulation, and altered nutrient harvest and availability—that favors the growth of pathogenic species while hindering the proliferation of beneficial microbes [9]. This represents a bidirectional relationship between chronic inflammation and dysbiosis, where microbial imbalances not only drive further inflammatory responses but also worsen clinical outcomes in various chronic diseases [10]. Therefore, understanding the role of gut microbiota in chronic inflammation among long-hauler patients is crucial for devising effective therapeutic strategies to alleviate symptoms and improve long-term personalized outcomes [9].

On the other hand, dietary interventions are recognized as significant modulators of inflammatory processes and microbial homeostasis in diverse disease scenarios [11]. Specifically, the Mediterranean diet (MD) has garnered considerable attention for multiple health benefits [12]. This dietary pattern is characterized by high consumption of fruits, vegetables, legumes, whole grains, nuts, and extra virgin olive oil (EVOO), which is rich in bioactive compounds such as polyphenols and omega-3 fatty acids, which underlie potent anti-inflammatory properties [13]. The consumption of EVOO, a cornerstone of the MD, is particularly noted for the high content of phenolic compounds with strong anti-inflammatory and antioxidant effects [14]. Numerous studies have consistently demonstrated that adherence to the MD is associated with reduced systemic inflammation markers, enhanced antioxidant defenses, and favorable alterations in gut microbiota composition and function [15]. Moreover, several investigations have elucidated the MD’s impact on gut microbiota diversity and functionality, indicating that the MD promotes the growth of beneficial microbial species while reducing the abundance of pro-inflammatory pathogens [16]. However, gaps remain in understanding how specific components of the MD, such as EVOO, modulate microbial communities, particularly in the context of chronic inflammatory conditions.

The objective of this research was to assess the influence of adherence to a Mediterranean diet on anthropometric, biochemical, and inflammatory variables, as well as the putative role of gut microbiota composition on such outcomes in participants with chronic inflammation. Additionally, this study aimed to explore the potential health benefits of antioxidant-rich food groups, such as extra virgin olive oil, in relation to inflammatory and metabolic conditions, with a particular focus on their interactive association with gut microorganisms.

## 2. Materials and Methods

### 2.1. Study Design

This study is part of the “METAINFLAMMATION” project (ref. Y2020/BIO-6600) that is a prospective and controlled study aimed at investigating the interaction concerning chronic inflammation and nutrition. Recruitment of participants took place between January 2022 and June 2023 at the Internal Medicine Service of Puerta de Hierro Majadahonda University Hospital in Madrid, Spain. Participants were included after providing informed consent, and the study adhered to the principles of the Declaration of Helsinki. Approval for the study protocol was granted by the Research Ethics Committee of Puerta de Hierro Majadahonda University Hospital (no. PI 164-21). Data collection strictly followed ethical guidelines and validated hospital protocols.

### 2.2. Participants, Inclusion, and Exclusion Criteria

This study enrolled a total of 188 adults, encompassing both men and women and all Caucasian and Hispanic descents. These participants presented a pathophysiological long-lasting chronic inflammation underlying a viral infection and followed the guidelines of the National Institute for Health and Care Excellence (NICE) and the National Institute for Health and Care Research (NIHR) [17,18]. This chronic inflammation was defined for this investigation as a prolonged inflammatory response due to immune dysregulation following the acute phase of the viral infection. Participants met the following inclusion criteria: age > 18 years, BMI > 17.01 kg/m^2^ and 51.35 kg/m^2^, and a diagnosis of long-COVID-19, confirmed by the medical staff of the Internal Medicine service of the Puerta de Hierro Majadahonda University Hospital (Madrid, Spain). These patients were initially diagnosed with COVID-19 through a combination of chest X-ray imaging and PCR testing to confirm the presence of the SARS-CoV-2 virus. Upon recovery from the acute phase of infection, they were later diagnosed with long COVID by the internist doctor, based on the persistence of mild COVID-19-like symptoms over an extended period. The treatments administered to patients were mainly symptomatic according to their long-COVID-19 presentation. For pain management, some individuals received analgesics, while inhalers were prescribed to those with some respiratory symptoms. Fatigue and general malaise were addressed through physical rehabilitation, facilitated by physiotherapy in certain cases, along with the use of acute pain relievers. Each therapeutic approach was carefully tailored to the patient’s unique clinical profile, ensuring that interventions were appropriate for their individual needs, according to the hospital protocols, but no one emerged as relevant, while corticoids only were administered in acute prescriptions and never in the previous four weeks in the recruited patients. In addition, they provided fecal samples, which were sequenced by a specialized external laboratory. The exclusion criteria included the presence of severe psychiatric disorders, the use of weight-modifying agents, pregnancy, or lactation, and the consumption of probiotics or antibiotics at least 3 weeks before the fecal sample collection, as well as the rejection to participate, as described elsewhere [19].

### 2.3. Anthropometrics Measurements

Anthropometric measurements, including body weight, height, waist circumference, and body composition using bioimpedance analysis, were conducted by qualified dietitians and clinical staff using validated methods and equipment, as describe elsewhere [19] Body weight was measured using a bioimpedance scale (TANITA SC-330; Tanita Corporation, Tokyo, Japan), which also provided body composition estimates. Waist circumference was assessed with a standard tape measure following established protocols performed by trained dietitians [20]. Body Mass Index (BMI) was calculated as the ratio of body weight to height squared (kg/m^2^), applying the international criteria set by the World Health Organization (WHO) (BMI < 24.9 kg/m^2^ for normal weight, BMI 25–29.9 kg/m^2^ for overweight, and BMI ≥ 30 kg/m^2^ for obesity) [21]. Body composition data derived from the TANITA SC-330 device (Tanita Corporation), as described elsewhere [19].

### 2.4. Lifestyle Variables

The baseline questionnaire of the METAINFLAMMATION cohort collected information on a wide array of characteristics previously validated including sociodemographic, lifestyle-related including age, sex, family history, smoking status, physical activity, prevalence of chronic diseases, napping, snacking habits, and dietary intake. Adherence to the Mediterranean dietary pattern was assessed using a well-known validated questionnaire for Spanish population [22,23]. The Supplementary Table S3 shows a detailed description of the questions and criteria to calculate the 14-MEDAS score [24].

### 2.5. Biochemical Data

Blood samples were collected under fasting conditions via venipuncture. These samples were analyzed for leukocytes, lymphocytes, neutrophils, hemoglobin, mean corpuscular volume, platelets, erythrocyte sedimentation rate (ESR), and erythrocyte distribution width (RDW) using an SYSMEX XN-20 automated hematology analyzer (Roche, Basel, Switzerland) [19]. The neutrophil/lymphocyte ratio was calculated directly from the measured values. Routine biochemical markers such as glucose, total cholesterol, glycated hemoglobin, uric acid, vitamin D, ferritin, gamma glutamyl transpeptidase (GGT), vitamin B12, folic acid, high-density lipoprotein (HDL), triglycerides, glutamic-oxaloacetic transaminase (GOT), and glutamic-pyruvic transaminase (GPT) were measured according to validated hospital protocols using a quality-controlled autoanalyzer (Atellica™ Solution, Siemens Healthineers, Erlangen, Germany) and established criteria. Prognosis-related variables, proinflammatory factors, and markers such as C-reactive protein (CRP), fibrinogen, insulin, lactate dehydrogenase (LDH), D-dimer, interleukin-6 (IL-6), and prothrombin activity were also assessed following standardized procedures, primarily utilizing ELISA kits (Sigma-Aldrich ELISA Kit, St. Louis, MO, USA) as per the suppliers’ instructions [19,25].

### 2.6. Metagenomic Analysis

Fecal samples were collected using OMNIgene^®^ · GUT kits (DNA Genotek, Ottawa, ON, Canada), following the manufacturer’s instructions. Bacterial DNA extraction was carried out with the QI-Aamp^®^ DNA kit (Qiagen, Hilden, Germany) as per the protocol provided. The V3-V4 hypervariable regions of the 16S rRNA gene were amplified using paired-end DNA sequencing on the MiSeq System (Illumina, San Diego, CA, USA) at Novogene Sequencing-Europe (Cambridge, UK). The PCR reactions utilized the following primers: 16S Amplicon PCR Forward Primer (5′-TCGTCGGCAGCGTCAGATGTGTATAAGAGACAGCCTACGGGNGGCWGCAG-3′) and 16S Amplicon PCR Reverse Primer (5′-GTCTCGTGGGCTCGGAGATGTGTATAAGAGACAGGACTACHVGGGTATCTAATCC-3′). Amplicon preparation was conducted using the 16S Metagenomic Sequencing Library Preparation Protocol (Illumina, San Diego, CA, USA), which includes overhang adapter sequences compatible with Illumina index and sequencing adapters. Amplicon size was confirmed by electrophoresis (LabChip GX; PerkinElmer, Waltham, MA, USA). The DNA libraries for 16S rRNA amplicon sequencing were prepared using the Nextera XT DNA Library Preparation Kit (Illumina, San Diego, CA, USA) following the manufacturer’s protocol. Sequencing of the 16S rRNA libraries was performed on the Illumina MiSeq benchtop sequencer in paired-end mode with 2 × 300 cycles using the MiSeq Reagent v3 600-cycle kit (Illumina, San Diego, CA, USA). Quality control included filtering of low-quality reads and removal of chimeric sequences post-alignment using the Quantitative Insights into Microbial Ecology program (QIIME2) [26,27]. The resulting clean reads were clustered into amplicon sequence variants (ASVs) using DADA2 and annotated with the SILVA v.132 16S rRNA gene database. Relative abundance of each ASV and alpha diversity were calculated using the Phyloseq R package [19,28,29]. Beta diversity was assessed using weighted UniFrac distances and visualized through principal coordinate analysis (PCoA). PERMANOVA was employed to compare similarity of bacterial communities among groups using the “vegan” package in R (version 2.5-7).

### 2.7. Statistical Analyses

Participants were stratified according to mean adherence to MD criteria using a validated questionnaire for a Spanish population (MEDAS14), being “lower adherence MD” (for those with values below or equal to 7) or “higher adherence MD” (for those with values above 7) for convenient statistical analyses. The selection of the cut-off was based on previous studies [23,24,30,31]. Variables were expressed as means and standard deviations for quantitative variables and the number of cases and percentage for qualitative variables. Student’s *t* tests were implemented to compare the means of the continuous variables at the beginning of the study, and the categorical variables were statistically analyzed using the chi-square (χ^2^) test. Correlation analyses were performed using Spearman and bivariate scatter plots for the representation. Potential interactions were investigated with general linear regression models that introduced the corresponding interaction terms into the models and using Stata 12. (StataCorp LLC, College Station, TX, USA). All analyses were performed adjusting by sex and age. The classification in antioxidant and prooxidant foods for the comparison by groups of adherence was performed according to the current scientific literature [32,33]. Consumption of EVOO was categorized by “low” and “high” using 3 servings/day as cut-off. A *p* value of <0.05 was considered statistically significant. Linear discriminant analysis (LDA) effect size (LEfSe) (http://huttenhower.sph.harvard.edu/galaxy/, accessed on 5 June 2024) was used to compare groups and visualize the results using taxonomic bar charts. Comprehensive differential abundance analyses were conducted using the MicrobiomeAnalyst web-based platform (https://www.microbiomeanalyst.ca/, accessed on 2 June 2024) [34]. Gene abundance data were analyzed by marker data profiling (MDP), filtering by a minimum count of 4, 20% prevalence in samples, and a low variance filter (maximum percentage of samples to remove) of 20%, based on inter-quantile range. Data were normalized using the centered-log ratio (CLR) using the zCompositions package for RStudio [35,36]. Alpha diversity profiling between groups with low and high inflammation scores was evaluated using the Shannon index. Beta diversity was assessed using Bray–Curtis distances and represented using principal coordinates analysis (PCoA) and the PERMANOVA test. Differential abundance was assessed using EdgeR as described (https://www.microbiomeanalyst.ca/, accessed on 2 June 2024) [34].

## 3. Results

### 3.1. Comparison of Anthropometrics, Biochemical, and Inflammatory Outcomes According to the Adherence to Mediterranean Diet

The stratification of the population, using 7 points as the cut-off from the MD adherence questionnaire, resulted in 54 participants with “lower adherence MD” (less than 7 points) and 134 participants with “high adherence MD” (higher than 7 points). The comparisons of anthropometric measurements and body composition according to MD adherence are shown in Table 1. Participants with high adherence to MD showed lower values in most of the anthropometric and body composition variables, with a marginally statistical difference. As expected, participants with high MD adherence had significantly higher adherence scores (Table 1).

In addition, Table S1 compared the prevalence of metabolic diseases in this cohort and sociodemographic and lifestyle variables between participants with low adherence and high adherence to the MD. Participants with high MD adherence had significantly lower prevalence of obesity (*p* = 0.04). Although not statistically significant, there was a trend towards a lower prevalence of diabetes mellitus in the high adherence group (*p* = 0.06). University education level was significantly higher in participants with higher adherence to MD, finding also more paid employees in this group. No significant differences were found between groups in the prevalence of hypertension, dyslipidemia, sadness, smoking status, or snacking habits.

Table 2 reports the comparison of biochemical and inflammatory outcomes based on the adherence to the MD. These findings highlight several biochemical benefits in the group with higher adherence to the MD. Indeed, participants with higher adherence showed lower total cholesterol and LDL-cholesterol levels compared to those with low MD adherence, suggesting improved lipid profile. In addition, volunteers with higher adherence presented lower glucose and insulin levels. Additionally, liver function markers were significantly better, with lower GOT levels observed in the higher adherence group. Moreover, LDH levels were lower in the high adherence group, which may reflect reduced tissue damage and oxidative stress. Although there were no significant differences in C-reactive protein and IL-6, high MD adherence was associated with higher folic acid levels and improved coagulation profiles, as evidenced by lower activated partial thromboplastin time (aPPT) and D-dimer levels. Noteworthy, recognized redox markers such as folic acid, glucose, LDL-cholesterol, LDH were significantly improved in the group with higher MD adherence.

### 3.2. Comparison of Food Consumption According to the Adherence to Mediterranean Diet

The Supplementary Table S2 shows a comparison of the reported frequency of consumption of several foods’ groups collected by the short validated FFQ, between groups of low and high adherence to the MD. The results indicated significant differences in the consumption of some foods between both groups. Participants with high adherence to the MD consumed white fish and fatty fish more frequently than those with low adherence. A higher consumption of vegetables, fruits and nuts was also observed in the high adherence group, while the consumption of lean meat and fatty meat did not show significant differences between the groups. In the case of whole and semi-skimmed dairy products, no significant differences were observed in the frequency of consumption between the groups. In addition, those with high adherence consumed legumes more frequently, with the majority reporting intake of 1–2 times per week or more. Olive oil usage was notably higher in the high adherence group, reflecting the significant role in the Mediterranean pattern. Although the use of other oils was present in both groups, it was significantly less frequent among those with high adherence. The consumption of refined grains was also higher among those with higher adherence, while whole grains were more frequently consumed by this group as well. No significant differences were observed in eggs consumption between groups. However, pastry and sugar consumption were significantly lower in the high adherence group, indicating a lower intake of sugary and processed foods. Alcohol consumption did not differ significantly between the groups, though a trend towards higher water intake was noted in the high adherence group.

**Figure 1:**
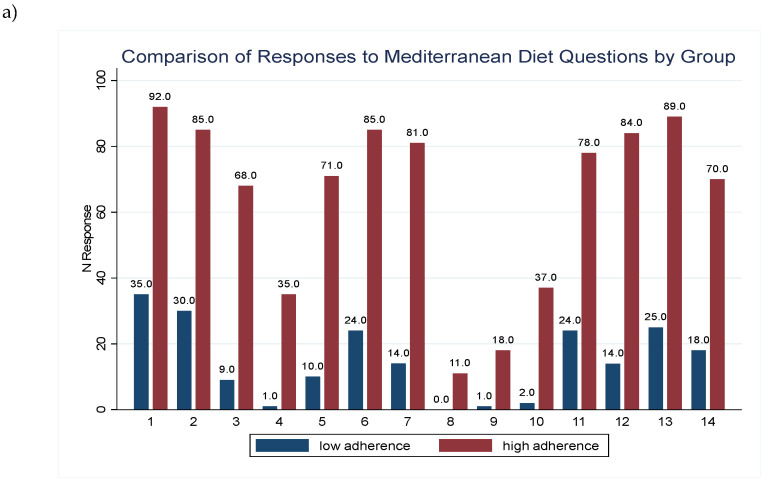

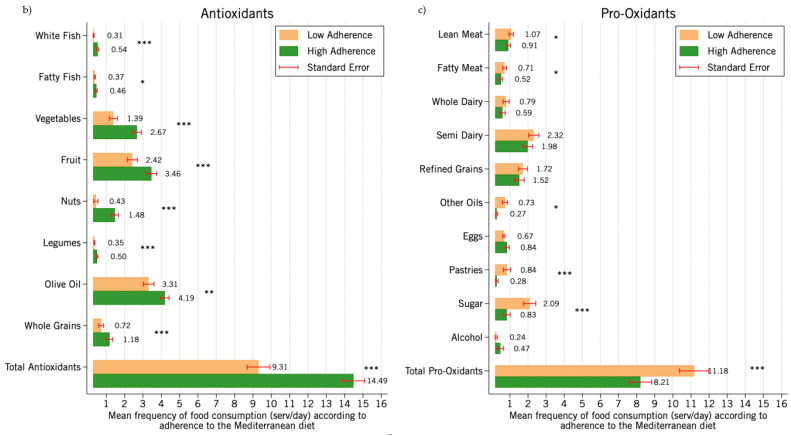
(**a**) Bar plot representing the N of positive response for each question in the Mediterranean Diet adherence questionnaire for both groups of comparison: low adherence (7 points, in blue) and high adherence (<7 points, in red). (**b**) Mean servings per day of antioxidant foods consumption according to adherence to the Mediterranean diet. (**c**) Mean servings per day of pro-oxidant foods consumption according to adherence to the Mediterranean diet. Yellow bars represent low adherence group (≤7 points) and green bars represent high adherence group (>7 points). Comparison was performed between low and high adherence groups, using Wilcoxon test * means *p* < 0.05, ** means *p* < 0.01 and *** means *p* < 0.001.

The Figure 1a shows the reported responses to the validated questionnaire of adherence to the MD for each group. Figure 1b,c shows the differences in the total mean of servings per day of food consumption between individuals with low adherence and high adherence to the MD. Figure 1a presents a bar plot illustrating the responses to each question in the MD adherence questionnaire, categorized by the level of adherence, showing that participants with higher adherence to the MD (red bars) reported more positive responses to each question. Specifically, for antioxidant-rich foods, participants with high adherence to the MD presented significantly higher consumption in white and fatty fish, vegetables, fruits, nuts, legumes, olive oil and whole grains (Figure 1b,c) [33]. In contrast, participants with low adherence to MD presented significantly higher values in the consumption of lean and fatty meat, other oils (different to olive oil), pastries and sugar (Figure 1b,c). Indeed, the total mean of servings per day for antioxidant intake was also significantly higher in the high adherence group (*p* < 0.001). In contrast, the mean of servings per day for pro-oxidant foods was significantly higher in participants with lower adherence to MD (*p* < 0.001).

### 3.3. Analysis of Gut Microbiota Diversity and Composition According to the Adherence to Mediterranean Diet

**Figure 2:**
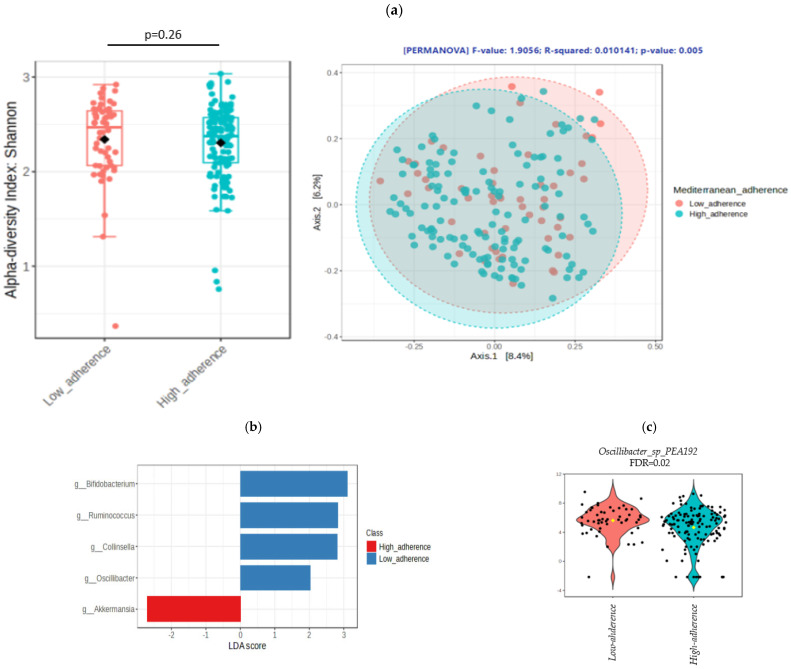
(**a**) Analysis of alpha and beta diversity by groups of adherence to Mediterranean diet. Box plot representing alpha diversity analysis evaluated by Shannon index according to low adherence to Mediterranean diet (orange box) and high adherence (blue box), compared using Wilcoxon test. Principal coordinate analysis for beta diversity calculated using Bray Curtis index and PERMANOVA. Red circles represent participants with low adherence to Mediterranean diet and blue circles represents participants with high adherence. (**b**) Analysis of the microbial structure based on linear discriminant analysis. Blue bars mean bacterial genus overrepresented in participants with low adherence to Mediterranean diet. Red bars mean bacterial genus overrepresented in participants with high adherence to Mediterranean diet. (**c**) Comparative analysis using EdgeR of *Oscillibacter* abundance according to groups of adherence to Mediterranean diet. Orange boxes represent participants with low adherence and blue boxes represent participants with high adherence. Yellow dot represents the mean value in each group of comparison. All *p* values were corrected by FDR.

The analysis of gut microbiota community profiling according to the adherence to MD is shown in Figure 2a. No significant differences were found between groups of comparison in alpha diversity assessed by Shannon index (*p* = 0.26). However, the analysis of beta diversity revealed significant differences between groups of adherence (*p* = 0.005). A comparative analysis of the taxonomic structure of the gut microbiota between these two groups of adherences was performed. Figure 2b shows a linear discriminant analysis of the differential abundance analysis performed to identify genus significantly different between groups of adherences. LEfSE analysis revealed that subjects with low adherence presented an overrepresentation of *Bifidobacterium*, *Ruminococcus*, *Collinsella*, and *Oscillibacter* genera. In contrast, participants with high adherence to MD showed an overrepresentation of *Akkermansia* genus. In addition, a comparative analysis of the abundance of *Oscillibacter* according to the adherence to MD was performed using EdgeR method (Figure 2c).

### 3.4. Analysis of Associations and the Interplay Between LDH and Oscillibacter According to the Adherence to Mediterranean Diet and the Consumption of Extra Virgin Olive Oil

**Figure 3:**
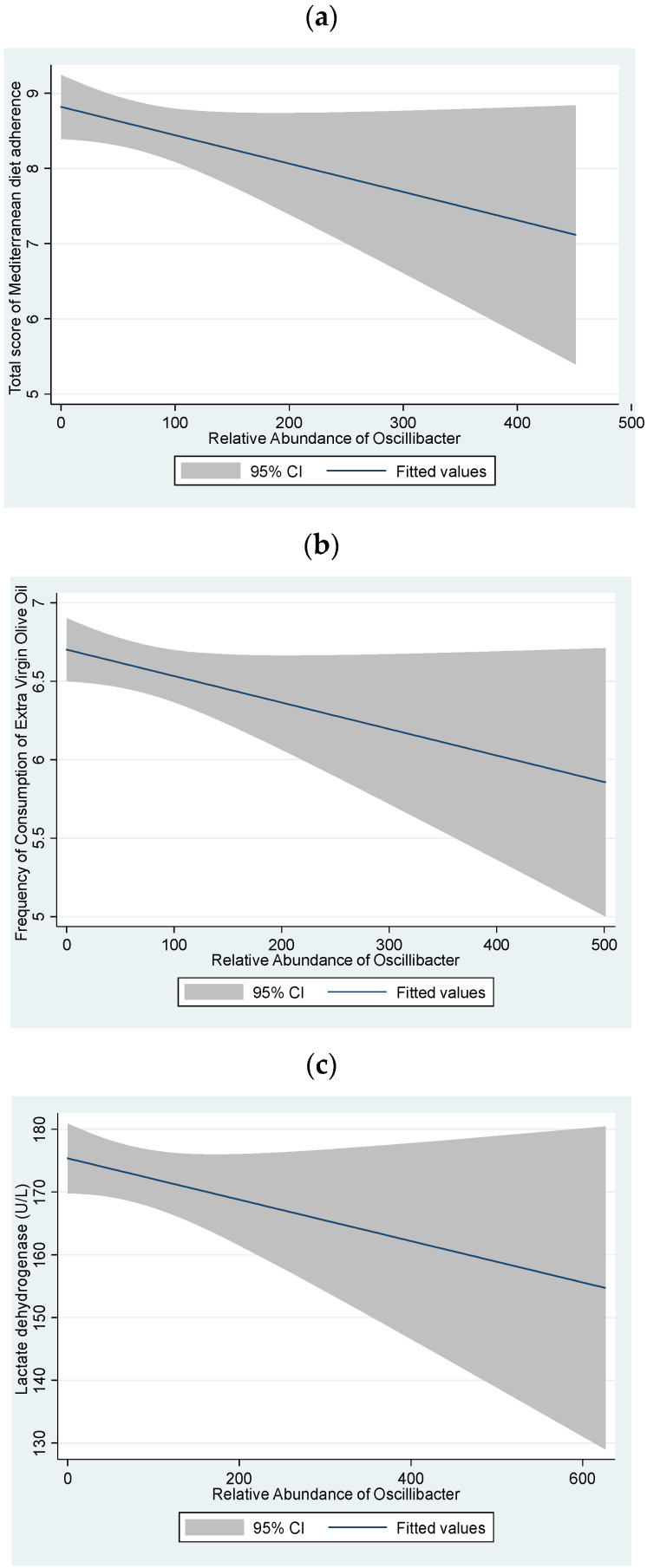
(**a**) Spearman correlation analysis between Mediterranean Diet score and *Oscillibacter* abundance (rho = −0.15, *p* = 0.04). The scatter plot illustrates the relationship between the Mediterranean Diet score (*y*-axis) and the abundance of Oscillibacter (*x*-axis) in participants. (**b**) Spearman correlation graph between *Oscillibacter* abundance and extra virgin olive oil consumption. The correlation coefficient (rho = −0.12, *p* = 0.04) indicates a significant inverse relationship, where higher *Oscillibacter* abundance is associated with lower consumption of extra virgin olive oil. (**c**) Spearman correlation analysis between LDH and *Oscillibacter* abundance (rho = −0.18, *p* = 0.01). The scatter plot illustrates the relationship between the *Oscillibacter* (*x*-axis) and LDH (*y*-axis) in participants of this cohort.

The analysis of the association between *Oscillibacter* abundance and adherence to Mediterranean diet resulted in a negative correlation (rho = −0.15, *p* = 0.04) (Figure 3a).

Figure 3b shows the negative association between *Oscillibacter* abundance and frequency of consumption of EVOO (rho = −0.12, *p* = 0.04). The association between LDH and *Oscillibacter* is shown in Figure 3c (rho = −0.18, *p* = 0.01).

Correlations heatmap were performed based on the adherence to the MD to assess the associations between food frequency consumption, biochemical markers, and gut microbiota composition. Similarly, Figure 3b shows the negative correlation found between *Oscillibacter* abundance and olive oil consumption (rho = −0.12, *p* = 0.04).

**Figure 4:**
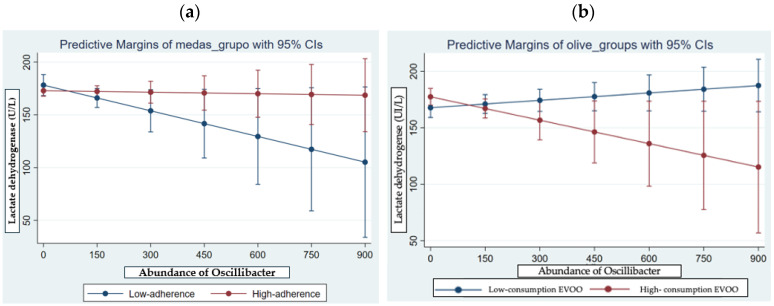
(**a**) Predicted values of LDH in long-haulers participants according to adherence to Mediterranean diet and consumption of extra virgin olive oil (categorized by more or less than 3 servings/day). (**b**) Models were adjusted by age, sex, and BMI.

An interaction model was performed to assess the relationship between consumption of EVOO, gut *Oscillibacter* and LDH. A significant interaction was observed between the abundance of *Oscillibacter*, the adherence to MD and LDH levels. The interaction plot (Figure 4a) reveals a trend where patients with a high abundance of *Oscillibacter*, together with a low adherence to MD, exhibit elevated LDH values. In other words, the presence of elevated levels of *Oscillibacter* appears to modulate the levels of LDH, according to the dietary adherence (R^2^ = 0.21; *p* = 0.03).

In addition, an analysis with the consumption of EVOO was performed in order to deep in the antioxidant compounds of the MD. The Figure 4b shows the significant interaction between *Oscillibacter* abundance, LDH levels and consumption of EVOO (categorized as more or less than 3 servings/day). The interaction plot illustrates the trend wherein patients exhibiting elevated abundance of *Oscillibacter* and low consumption of EVOO, presented higher predicted values of LDH (R^2^ = 0.14; *p* = 0.02).

## 4. Discussion

The main objective of this research was to evaluate the adherence of MD impact on anthropometric, biochemical, inflammatory outcomes, and gut microbiota composition in patients with long-hauler chronic inflammation. Additionally, this study aimed to assess the interplay between gut bacteria and a biomarker of oxidative stress (LDH) with an antioxidant food group (EVOO), typically presented in the Mediterranean pattern.

The dietary pattern analyzed in this study, marked by a higher intake of fruits, vegetables, nuts, fish, and olive oil, is consistent with the diet’s core principles and the well-established health benefits. These foods are rich sources of antioxidants, polyphenols, and omega-3 fatty acids, which collectively contribute to the MD’s anti-inflammatory and cardioprotective effects [33,37,38]. The lower intake of refined grains, sugars, and other oils in the high adherence group further supports the MD diet’s role in reducing systemic inflammation and improving metabolic health [39]. Indeed, previous studies have demonstrated that individuals with metabolic syndrome who adhered closely to the MD exhibited more favorable anthropometric measurements, improved blood biochemical profiles, and better oxidative and inflammatory markers compared to those with metabolic syndrome who had lower adherence to the MD [40]. These findings underline the beneficial impact of the MD on chronic diseases and are in agreement with previous investigations [41,42].

On the other hand, some studies have shown that gut microbiota in severe COVID-19 cases was significantly altered compared to healthy controls, highlighting the potential implications for immune response and inflammation [43,44]. Additionally, Grosso et al. (2017) reviewed the effects of the MD, describing the anti-inflammatory benefits and its potential to lower inflammatory markers through increased adherence [45]. However, to our knowledge, no studies have specifically investigated the effects of the MD on patients with persistent COVID-19 and chronic inflammation, particularly with an integrated analysis of metagenomic, anthropometric, biochemical, and inflammatory outcomes data and the antioxidant components of the MD. By integrating dietary intake data with microbiome analyses, this study sought to elucidate novel mechanisms through which EVOO and the MD may mitigate inflammation and improve gut health in long-hauler patients by highlighting the role of the MD—a diet rich in antioxidants and bioactive compounds—in modulating key health biomarkers.

The analysis performed in this investigation revealed differences between individuals with low and high adherence to the MD in key health indicators. Although the BMI difference between groups was not statistically significant, the trend towards a lower BMI in the high adherence group supports the existing literature indicating potential benefits of MD on weight management [46,47,48]. The observed trends in waist circumference and waist/height ratio suggested a beneficial impact of MD on central adiposity, although not reaching statistical significance. Overall, there was a general trend indicating that adherence to the MD was associated with improvements in certain anthropometric measures, such as waist circumference and waist/height ratio, which may reflect a positive impact on central obesity and overall body composition, in accordance with existing data [49,50].

On the other hand, biochemical markers showed significant improvements in participants with high adherence to the MD, with reductions in total cholesterol, LDL-cholesterol, glucose, insulin levels, GOT, folic acid, apTT, and LDH.

Total cholesterol showed a significant reduction in participants with adherence to MD. In this context, the scientific literature reported that MD can improve levels of cholesterol, specifically high-density lipoproteins (HDLs), contributing to the cardioprotective role of the Mediterranean pattern. In this investigation, participants with higher adherence showed higher levels of HDL-cholesterol, although without significant difference. However, LDL-cholesterol presented a significant reduction in participants with high adherence. In this sense, the study of Hernáez et al. found that the adherence to the MD, particularly when enriched with virgin olive oil, decreased LDL atherogenicity in high cardiovascular risk individuals [51]. In this line, Meslier et al. showed that people with obesity who followed a MD reduced their blood cholesterol and caused multiple changes in their microbiome and metabolome, improving metabolic health [52].

Inflammation and disruptions in lipid metabolism are related to the development of atherosclerosis [53]. LDL oxidation has been identified as a key atherogenic modification within the vascular endothelia. Recent research has expanded this view, indicating that LDL particles undergo various modifications affecting their size, density, and chemical properties. Oxidation represents a terminal stage in this modification process, contributing to LDL’s atherogenic potential. Significantly, oxidized LDL (oxLDL) has been recognized for a complex role in inflammation, displaying pro-inflammatory properties. Elevated oxLDL levels are known to activate macrophages, which can aggravate inflammatory responses, especially in the context of chronic inflammatory conditions such as persistent COVID-19. Given the persistent inflammatory state seen in these individuals, controlling LDL oxidation is vital for mitigating inflammatory processes [54].

In addition, glucose levels showed a significant reduction in participants with high MD adherence. Elevated glucose levels are known to be prooxidative, contributing to increased oxidative stress and exacerbating inflammatory conditions [55]. In the context of persistent chronic inflammation, high glucose levels can further intensify oxidative damage and inflammation [56]. By contrast, a lower glucose profile, as seen in those with high adherence to the MD, may mitigate these adverse effects. This finding evidenced that MD’s adherence impact on reducing glucose levels could therefore play a crucial role in lowering oxidative stress and inflammation.

Moreover, insulin levels showed a significant reduction in the high adherence group. Elevated circulating insulin levels are a hallmark of insulin resistance, which is closely linked to increased oxidative stress and inflammation [57]. Specifically, hyperinsulinemia can exacerbate oxidative damage by promoting the production of ROS and enhancing the inflammatory response through signaling pathways, including the activation of nuclear factor kappa B (NF-kB) and the inflammasome [58].

Thus, in this sense, MD, characterized by a low glycemic index and high content of anti-inflammatory and antioxidant foods, contributes to decreased insulin levels, improved insulin sensitivity, and reduced activation of the inflammatory pathways, thereby mitigating systemic inflammation [59]. This is particularly relevant for patients with chronic inflammatory conditions, as reduced insulin levels can help alleviate the oxidative and inflammatory sequelae associated with these conditions.

GOT showed a significant decrease in participants with high adherence to MD. Elevated GOT levels are often indicative of increased oxidative damage and inflammation within the liver and other tissues, reflecting a state of chronic inflammation or metabolic dysregulation [60]. In this context, persistent elevation of GOT is often associated with ongoing liver inflammation and increased oxidative stress, both of which are exacerbated by chronic inflammatory conditions [61].

In addition, participants with high adherence to MD presented significantly higher levels of folic acid. Folic acid is a vital B vitamin and plays a crucial role in one-carbon metabolism, which is essential for DNA synthesis, repair, and methylation, necessary to maintain cellular homeostasis and mitigate oxidative stress [62]. Deficiencies in folic acid are associated with increased homocysteine levels, which can lead to elevated oxidative stress and inflammation, further exacerbating cardiovascular risk and metabolic disorders. The MD, rich in folate-containing foods such as leafy greens, legumes, and whole grains, increases folic acid levels, thereby potentially reducing oxidative damage and metabolic health [63]. Similarly, the reduction in aPTT indicates improvements in coagulation status and vascular health. Prolonged aPTT can be indicative of coagulation disorders or increased risk of bleeding, while normalization or reduction in aPTT suggests better regulation of the coagulation cascade [64].

Among the observed biomarkers, the reduction in LDH levels in participants with high adherence to the MD was particularly significant. The MD, with a high content of antioxidant-rich foods, can help decrease cellular damage and consequently lower LDH levels [65]. LDH is an enzyme involved in the conversion of lactate to pyruvate and vice versa, and elevated levels are often used as an indirect marker of tissue damage and oxidative stress [6]. High circulating levels of LDH can indicate increased cellular injury and heightened oxidative stress, as it is released into the bloodstream following cellular damage and inflammation. In the context of persistent chronic inflammation and oxidative stress, which are prevalent, the reduction in LDH levels among those adhering to the MD reflects an improvement in oxidative balance and a reduction in systemic inflammation. Lower LDH levels in this cohort suggest a decreased burden of oxidative damage and a potential improvement in cellular health. This is particularly relevant given the elevated oxidative stress and tissue damage often observed in patients with persistent COVID-19 [66].

On the other hand, the analysis of gut microbiota composition revealed significant differences in beta diversity between high and low MD adherence groups, with high adherence associated with a more favorable gut microbial profile, including increased abundance of *Akkermansia*, a genus linked to improved gut barrier function and reduced inflammation [67]. Current evidence suggests that the MD’s rich fiber and polyphenol content can positively modulate gut microbiota, promoting the growth of beneficial bacteria while suppressing pathogenic species [68]. Among the microbiota taxa of interest, *Oscillibacter* has emerged as a noteworthy genus with potential implications for dietary interventions and inflammatory conditions [69,70]. This genus is known for the potential to produce short-chain fatty acids (SCFAs) such as butyrate, which have been shown to exert anti-inflammatory effects and improve gut barrier function [71]. Although there is limited research linking *Oscillibacter* with adherence to the MD, emerging studies provide insight into how dietary components might influence this microbial genus. The MD, characterized by its high fiber content and bioactive compounds, has the potential to impact *Oscillibacter* populations, which are known for their role in fiber fermentation and the production of SCFAs beneficial for intestinal health. Dietary fiber could promote conditions favorable to the growth of *Oscillibacter*, although the specific effects of the MD were not directly addressed in their study [71]. Additionally, *Oscillibacter* has been linked to various metabolic and health effects. It has been suggested that this genus may play a role in modulating inflammation and metabolic health and might be associated with inflammatory states [72]. Although their study did not focus on the MD, these findings imply that the interaction between *Oscillibacter* and dietary patterns like the MD could influence the bacteria’s impact on inflammation. The MD, known for its anti-inflammatory properties, might therefore interact with *Oscillibacter* in ways that could affect systemic inflammation and overall health. Further research is needed to clarify these relationships and their implications for dietary interventions.

Several investigations have indicated that SCFAs, including those produced by *Oscillibacter*, participate in the regulation of immune responses and reduction of inflammation. These microbiota-derived metabolites help to maintain the integrity of the gut lining, modulate T-cell function, and inhibit the production of pro-inflammatory cytokines [73]. Therefore, an increase in *Oscillibacter* because of MD adherence could contribute to a reduction in inflammatory markers and improve clinical outcomes in patients with persistent inflammatory conditions, such as long-hauler patients.

A major finding of this research was the interactive associations between LDH levels and the abundance of the gut microbiota genus *Oscillibacter*. The Oscillospiraceae family and *Oscillibacter* genus have been associated with leanness and reduced inflammation, suggesting a beneficial role in metabolic health [74]. This analysis found that individuals with high MD adherence had a more favorable gut microbiota composition, including higher levels of Oscillibacter, which inversely correlated with lower LDH levels. This suggests a potential link between diet, gut health, and systemic inflammation, where the MD fosters a gut environment that supports beneficial bacteria like *Oscillibacter*, which in turn may contribute to lower oxidative stress markers such as LDH.

Precision nutrition strategies could leverage these insights by incorporating dietary components known to promote beneficial bacteria abundance and food groups rich in polyphenols and antioxidant properties from the MD. By targeting gut microbiota modulation through personalized MD interventions, it may be possible to enhance antioxidant defenses and reduce systemic inflammation, as indicated by biomarkers like LDH, which are potent for precision nutrition.

Olive oil, particularly extra virgin olive oil, is a key component of the MD and is well-documented for its rich content of monounsaturated fats and bioactive compounds, including polyphenols like oleocanthal and hydroxytyrosol [14]. These compounds are potent antioxidants and have been shown to exert anti-inflammatory, cardioprotective, and neuroprotective effects. Comparative studies have demonstrated that olive oil’s antioxidant properties significantly reduce oxidative stress markers, including LDH, and improve lipid profiles, as seen with reductions in total cholesterol and LDL-cholesterol [75,76]. Furthermore, EVOO’s ability to enhance the bioavailability of fat-soluble vitamins and polyphenols from other MD components further amplifies the health benefits, making it a key element in precision nutrition strategies aimed at reducing oxidative stress and inflammation [77]. Several studies have demonstrated that polyphenols from EVOO can modify the gut microbiome by enhancing microbial diversity and promoting beneficial bacterial growth [78,79]. While these studies did not specifically focus on *Oscillibacter*, the general finding that EVOO influences gut microbiota composition suggests that it could affect *Oscillibacter* abundance. The interaction between EVOO consumption, LDH levels, and gut microbiota composition, including *Oscillibacter*, highlights a complex interplay where diet influences systemic health through multiple pathways. This integrated view aligns with current research suggesting that dietary antioxidant components can modulate gut microbiota composition, enhance gut barrier function, and reduce systemic inflammation [80,81].

Furthermore, the interaction between *Oscillibacter* and EVOO consumption has implications for systemic inflammation. The presence of EVOO in the diet has been linked to lower levels of inflammatory markers, potentially moderated by its effects on the gut microbiota. For example, a study by Wongwarawipat et al. (2017) [82] found that EVOO consumption was associated with reduced inflammation and improved metabolic markers, which could be partly mediated by changes in gut microbiota composition, including *Oscillibacter*. This study provides a thorough examination of the relationship between *Oscillibacter*, adherence to the MD, and oxidative stress markers like LDH, evidencing that LDH outcomes affect *Oscillibacter* but depend on MD adherence and EVOO consumption.

In addition, the analysis incorporated specific Mediterranean antioxidant food groups (EVOO) and enhances the analysis by evaluating the effects of a key MD component on gut microbiota and inflammation. Thus, these findings provide novel insights into the potential of the MD to reduce inflammatory markers specifically in patients with long-COVID-19, a population that has scarcely investigated concerning dietary patterns. Furthermore, our findings introduce a previously unexplored role of *Oscillibacter* as a modulating factor in the relationship between dietary adherence and inflammatory outcomes. This highlights the potential for microbiota-mediated mechanisms to influence chronic inflammation in post-viral conditions, offering a new perspective in the field of personalized nutritional interventions and microbiome research. Nevertheless, some limitations should be considered. The sample size may also limit the generalizability of the findings; thus, expanding the cohort to include a larger and more diverse population would enhance the robustness and applicability of the results. Additionally, the current study’s dietary assessment does not encompass all components of the MD. Future studies should incorporate a more comprehensive evaluation of MD elements to fully understand their influence on gut microbiota and inflammation, including frequency of consumption but also quantity.

Another aspect of this study is that pulmonary function was assessed by protocolized X-ray imaging and classical inflammatory measurements. Thus, inflammatory proxies such as CRP and IL-6 were measured, as well as indirect markers such as ESR, LDH, and fibrinogen, which can serve as valuable indicators of systemic inflammatory burden, which often reflects or correlates with pulmonary inflammation [2]. In fact, elevated LDH has been associated with tissue injuries and inflammation, including damage to lung tissue, while fibrinogen plays a central role in both systemic inflammation and clotting processes, which may also impact lung function in inflammatory conditions. Furthermore, it is important to emphasize that these participants are no longer carriers of the virus, but they continue to experience symptoms due to immune dysregulation, resulting in chronic inflammation. In addition, all patients underwent a systematic X-ray examination since they were diagnosed with coronavirus, which also ensured that the recruitment group was homogeneous, where no critically ill subjects were encountered affecting the lungs.

While additional variables, such as cultural and religious factors, could have further enriched, this study has incorporated key sociodemographic variables, including educational level and employment status, alongside the prevalence of chronic diseases (Table S1), providing insights into potential influences on dietary adherence and a more contextualized view of the study population. Additionally, in this study, a cut-off value of 7 was used to stratify participants into low and high adherence to the Mediterranean diet. This cut-off was selected based on previous studies utilizing the validated MEDAS14 questionnaire, where a score of 7 has been shown to be a reliable threshold for differentiating adherence levels in the Spanish population [23,24,30,31]. The use of this cut-off facilitates comparisons with other studies on Mediterranean diet adherence, although a different threshold can be determined for future studies and exploring alternatives.

Finally, by incorporating these insights into precision nutrition frameworks, dietary interventions can be designed to not only meet individual macronutrient, and micronutrient needs but also to optimize gut health and systemic inflammation markers [83]. For example, individuals with dysbiosis or elevated LDH levels might receive personalized dietary advice emphasizing a higher intake of olive oil and specific polyphenol-rich foods known to support beneficial gut bacteria and reduce oxidative stress.

These tailored dietary strategies not only enhance the MD’s health benefits but also support broader precision nutrition goals of optimizing gut health as a pathway to improving systemic inflammation, metabolic function, and immune response [84]. Future research should focus on further elucidating the complex interactions between the MD, gut microbiota, and host health, with an emphasis on identifying specific dietary components that drive beneficial microbial shifts. In addition, future research should incorporate advanced omics technologies and integrative approaches to better understand how genetic, epigenetic, and environmental factors influence dietary outcomes. Such insights will be crucial for refining precision nutrition strategies and developing more effective, individualized dietary interventions that leverage the MD’s rich array of antioxidant compounds and functional foods.

## 5. Conclusions

This study demonstrates that long-hauler patients with high adherence to the Mediterranean diet (MD) presented significant health benefits, showing better values in oxidative stress biomarkers such as LDH and featuring a lower abundance of gut *Oscillibacter*. Additionally, patients with low MD adherence or low EVOO consumption, coupled with high *Oscillibacter* abundance, presented lower LDH levels, suggesting a potential link between dietary patterns, microbial composition, and inflammatory responses. These approaches seek to contribute to the development of personalized dietary strategies that can effectively manage inflammation and enhance health outcomes for long-hauler patients.

## Figures and Tables

**Table 1:** Comparison of anthropometric measurements, body composition, and typical characteristics between post-viral long-hauler patients categorized by adherence to Mediterranean diet (more than 7 points and lower than 7) in METAINFLAMATION cohort.

Variables	Low Adherence MD (≤7 Points) (n = 54)	High Adherence MD (>7 Points) (n = 134)	*p* Value *
Gender (female)	47 (87.0)	107 (79.8)	**0.03**
Age (y)	49 ± 0.9	52 ± 0.8	**0.02**
Body Mass Index (kg/m^2^)	30.2 ± 0.9	28.4 ± 0.5	0.08
Waist (cm)	102 ± 2.0	98 ± 1.3	0.08
Waist/height ratio	0.6 ± 0.01	0.5 ± 0.01	0.06
Fat free mass (kg)	46.1 ± 1.0	46.6 ± 0.7	0.72
Fat mass (kg)	37.9 ± 1.3	35.4 ± 0.8	0.11
Visceral fat (AU)	9.5 ± 0.6	9.5 ± 0.4	0.97
Bone mass (kg)	2.4 ± 0.05	2.4 ± 0.03	0.77
Systolic pressure (mmHg)	121.3 ± 2.1	125.8 ± 1.5	0.10
Diastolic pressure (mmHg)	79.2 ± 1.5	76.9 ± 0.9	0.21
Physical activity (METs-min/w)	1114 ± 256	1399 ± 324	0.31
Mediterranean adherence	5.4 ± 0.1	9.8 ± 0.07	**<0.001**

Data was presented as mean ± standard deviation and *p* values. The significance threshold was set at *p* < 0.05 *, *t*-test was used to compare the mean of continuous variables and chi-square (χ^2^) to compare categorical variables. Lower adherence to Mediterranean diet refers to less than 7 points in the questionnaire and high adherence refers to more than 7 points. *p* value column is the comparison of variables’ mean between two categories of adherence using *t*-test or Mann–Whitney test, according to the distribution of the data. AU: arbitrary units; MET, metabolic equivalent task. *p* value lower than 0.05 in bold type.

**Table 2:** Comparison of biochemical and inflammatory outcomes according to the adherence to Mediterranean diet in METAINFLAMATION cohort.

Variables	Low Adherence MD (≤7 Points) (n = 54)	High Adherence MD (>7 Points) (n = 134)	*p* Value *
Uric acid (mg/dL)	4.8 ± 0.1	5.1 ± 0.1	0.12
Total cholesterol (mg/dL)	203 ± 3	189 ± 4	**0.01**
Triglycerides (mg/dL)	107 ± 7.6	103 ± 4.7	0.67
LDL-cholesterol (mg/dL)	121 ± 3	110 ± 4	**0.04**
HDL-cholesterol (mg/dL)	58.7 ± 2.1	60.2 ± 1.4	0.59
Glycated hemoglobin	5.4 ± 0.08	5.3 ± 0.03	0.48
Glucose (mg/dL)	111.3 ± 4.1	99.0 ± 3.8	**0.03**
Insulin (µU/mL)	11.7 ± 1.1	8.0 ± 0.8	**0.03**
GOT (U/L)	23.7 ± 0.7	19.9 ± 0.7	**<0.001**
GPT (U/L)	25.4 ± 1.2	22.7 ± 1.1	0.21
GGT (U/L)	26.5 ±1.9	25.2 ± 2.1	0.71
Vitamin D (nmol/L)	66.9 ± 6.3	76.7± 5.2	0.28
Lactate dehydrogenase (U/L)	177.2 ± 2.7	168.2 ± 3.6	**0.04**
C-reactive protein (mg/L)	2.6 ± 0.4	2.7 ± 0.3	0.91
IL-6 (pg/mL)	2.4 ± 0.5	2.5 ± 0.2	0.75
Ferritin (ng/mL)	90.3 ± 13.6	94.7 ± 6.1	0.72
Fibrinogen (mg/dL)	382.6 ± 9.9	335.2 ± 6.0	0.86
Vitamin B12 (pg/mL)	531.4 ± 34.1	502.7 ± 20.0	0.45
Folic acid (ng/mL)	6.4 ± 0.5	7.8 ± 0.3	**0.02**
Leukocytes (×10^3^/μL)	6.0 ± 0.2	6.2 ± 0.1	0.48
Neutrophils (×10^3^/μL)	3.5 ± 0.2	3.6 ± 0.1	0.48
Lymphocytes (×10^3^/μL)	1.9 ± 0.08	1.9 ± 0.05	0.56
Neutrophils/lymphocyte ratio	1.84 ± 0.08	1.89 ± 0.07	0.78
Platelets (×10^3^/μL)	266.5 ± 7.7	264.3 ± 5.6	0.82
Hemoglobin (g/dL)	14.4 ± 0.1	14.4 ± 0.09	0.65
RDW (%)	13.4 ± 0.07	13.2 ± 0.1	0.44
ESR (mm/h)	10.2 ± 0.9	11.2 ± 0.5	0.38
Prothrombin activity (%)	105.6 ± 1.3	100.4 ± 3.2	0.08
aP thromboplastin time (s)	37.1 ± 3.8	30.4 ± 0.3	**0.04**
D-dimer (ng/mL)	379 ± 25.5	332.8 ± 35.1	**0.04**

Data are presented as mean ± standard deviation and *p* values. The significance threshold was set at *p* < 0.05 *. Lower adherence to Mediterranean diet refers to less than 7 points in the questionnaire and high adherence refers to more than 7 points. *p* value column is the comparison of groups according to the adherence; ESR, erythrocyte sedimentation rate; GOT, glutamic-oxaloacetic transaminase; GPT, glutamic-pyruvic transaminase; GGT, gamma-glutamyl transferase; IL-6, interleukin-6; RDW, red cell blood distribution width. *p* value lower than 0.05 in bold type.

## Data Availability

The data presented in this study are available on request from the corresponding author due to privacy concerns and ethical restrictions. Data sharing will be considered upon reasonable request and under the condition of an approved data-sharing agreement once the whole project is finished.

## Supplementary Materials

The following supporting information can be downloaded at: https://www.mdpi.com/article/10.3390/antiox13111358/s1.
