# Supplementary material for: Mediterranean Diet and Olive Oil Redox Interactions on Lactate Dehydrogenase Mediated by Gut Oscillibacter in Patients with Long-COVID-19 Syndrome
Source: Antioxidants (Basel). 2024 Nov 6;13(11):1358. doi: 10.3390/antiox13111358 (PMC11591431; doi:10.3390/antiox13111358)
Supplement: Supplementary file 1 [file antioxidants-13-01358-s001.zip › antioxidants-3262710-supplementary.pdf]

Supplementary material

Table S1. Comparison of prevalence of metabolic diseases, sociodemographic and lifestyle variables related with the Mediterranean pattern, between post-viral long hauler patients categorized by adherence to Mediterranean diet in METAINFLAMATION cohort.

**Variables**	**Low adherence MD ((≤ 7 points) (n= 54)**	**High adherence MD (> 7 points) (n= 134)**	**P value**
Education (n, %)			
Primary education	3 (5.5)	3 (2.2)	
Secondary education	5 (9.2)	17 (12.7)	
Vocational training	23 (42.6)	26 (19.4)	
University education	23 (42.6)	87 (64.9)	
Other	0	1 (0.7)	
			**0.01**
Occupation (n, %)			
Full-time studies	2 (3.7)	2 (1.5)	
Paid employment	31 (57.4)	81 (60.4)	
Unemployment	1 (1.8)	17 (12.7)	
Permanent incapacity due to	18 (33.3)	22 (16.4)	
illness	0 (0)	9 (6.7)	
Retired	0 (0)	3 (2.2)	
Homemaker	2 (3.7)	0 (0)	
Other			
			**0.002**
Diagnosis of obesity (n, %)			
No	35 (64.8)	106 (79.1)	
Yes	19 (35.2)	28 (20.9)	
			**0.04**
Diagnosis of diabetes mellitus (n, %)			
No	47 (87.0)	127 (94.8)	
Yes	7 (12.9)	7 (5.2)	
			0.06
Diagnosis of hypertension (n, %)			
No	46 (85.1)	106 (79.1)	
Yes	8 (14.8)	28 (20.9)	
			0.33
Diagnosis of dyslipidemia (n, %)			
No	40 (74.1)	99 (73.9)	
Yes	14 (25.9)	35 (26.1)	
			0.97
Sadness (n, %)			
No	10 (18.5)	30 (22.4)	
Yes	44 (81.5)	104 (77.6)	
			0.55

Smoking (n, %)			
No	34 (62.9)	86 (64.2)	
Yes	3 (5.5)	7 (5.2)	
Ex-smoker	17 (31.5)	41 (30.6)	
			0.98
Snacking habit (n, %)			
No	27 (50.0)	52 (38.8)	
Yes	27 (50.0)	82 (61.2)	
			0.15
Nap (n, %)			
No	22	58	
Yes	32	76	
			**0.04**

Data was presented as n (standard deviation). Lower adherence to Mediterranean diet refers to less than 7 points in the questionnaire and high adherence refers to more than 7 points. P value column is the result of Chi2 test. P value lower than 0.05 in bold type.

Table S2. Comparison of reported frequency of food consumption in METAINFLAMMATION cohort based on adherence to the Mediterranean diet.

Variables	Low adherence MD (n= 54)	High adherence MD (n= 134)	P value
White fish			
Never or almost never	7 (63.4)	4 (36.3)	
1 to 3 times per month	16 (40)	24 (60)	
1 or 2 times per week	29 (30.5)	66 (69.4)	
3 or 4 times per week	2 (6.6)	28 (93.3)	
5 or 6 times per week	0 (0)	8 (100)	
Once a day	0 (0)	4 (100)	
2 or 3 times per day	0 (0)	0 (0)	
4 or 5 times per day	0 (0)	0 (0)	
6 or more times per day	0 (0)	0 (0)	
			**<0.001**
Fat fish			
Never or almost never	9 (16.36)	2 (1.5)	
1 to 3 times per month	9 (16.6)	25 (18.6)	
1 or 2 times per week	30 (55.5)	78 (58.2)	
3 or 4 times per week	4 (7.4)	25 (18.6)	
5 or 6 times per week	2 (3.7)	3 (2.2)	
Once a day	0 (0)	1 (0.7)	
2 or 3 times per day			
4 or 5 times per day			
6 or more times per day			
			**<0.001**
Lean meat			
Never or almost never	0 (0)	3 (2.2)	
1 to 3 times per month	0 (0)	8 (6.0)	
1 or 2 times per week	10 (18.5)	40 (29.8)	
3 or 4 times per week	28 (51.8)	52 (38.8)	
5 or 6 times per week	7 (13.0)	14 (10.4)	
Once a day	8 (14.8)	3 (2.2)	
2 or 3 times per day	1 (1.8)	0 (0)	
4 or 5 times per day	0 (0)	0 (0)	
6 or more times per day	0 (0)	0 (0)	
			0.19
Fat meat			
Never or almost never	6 (11.1)	17 (12.7)	
1 to 3 times per month	7 (13.0)	22 (16.4)	
1 or 2 times per week	15 (27.8)	61 (45.5)	
3 or 4 times per week	17 (31.5)	21 (15.7)	
5 or 6 times per week	5 (9.2)	8 (6.0)	
Once a day	3 (5.5)	4 (3.0)	
2 or 3 times per day	1 (1.8)	1 (0.7)	
4 or 5 times per day	0 (0)	0 (0)	
6 or more times per day	0 (0)	0 (0)	
			0.13

Vegetables			
Never or almost never	5 (9.2)	2 (1.5)	
1 to 3 times per month	10 (28.5)	9 (6.7)	
1 or 2 times per week	16 (29.6)	20 (14.9)	
3 or 4 times per week	11 (20.3)	20 (14.9)	
5 or 6 times per week	5 (9.2)	29 (21.6)	
Once a day	7 (12.8)	53 (39.5)	
2 or 3 times per day	1 (1.8)	1 (0.7)	
4 or 5 times per day	0 (0)	0 (0)	
6 or more times per day	0 (0)	0 (0)	
			**<0.001**
Fruits			
Never or almost never	3 (5.5)	2 (1.5)	
1 to 3 times per month	0 (0)	8 (6.0)	
1 or 2 times per week	14 (25.9)	4 (3.0)	
3 or 4 times per week	6 (11.1)	6 (4.5)	
5 or 6 times per week	1 (1.8)	10 (7.4)	
Once a day	8 (14.8)	27 (20.1)	
2 or 3 times per day	22 (40.7)	64 (47.8)	
4 or 5 times per day	0 (0)	13 (9.7)	
6 or more times per day	0 (0)	0 (0)	
		0 (0)	**<0.001**
Whole dairy			
Never or almost never	25 (46.3)	81 (60.4)	
1 to 3 times per month	7 (12.9)	9 (6.7)	
1 or 2 times per week	6 (11.1)	18 (13.4)	
3 or 4 times per week	3 (5.5)	4 (3.0)	
5 or 6 times per week	1 (1.8)	1 (0.7)	
Once a day	10 (18.5)	17 (12.7)	
2 or 3 times per day	2 (3.7)	4 (3.0)	
4 or 5 times per day	0 (0)	0 (0)	
6 or more times per day	0 (0)	0 (0)	
			0.49
Semi dairy			
Never or almost never	9 (16.7)	43 (32.1)	
1 to 3 times per month	3 (5.5)	5 (3.7)	
1 or 2 times per week	5 (9.2)	4 (3.0)	
3 or 4 times per week	4 (7.4)	7 (5.2)	
5 or 6 times per week	0 (0)	5 (3.7)	
Once a day	17 (31.5)	35 (26.1)	
2 or 3 times per day	15 (27.8)	33 (24.6)	
4 or 5 times per day	0 (0)	2 (1.5)	
6 or more times per day	1 (1.8)	0 (0)	
			0.11

Nuts			
Never or almost never	20 (37.0)	8 (5.9)	
1 to 3 times per month	17 (31.5)	8 (5.9)	
1 or 2 times per week	6 (11.1)	20 (14.9)	
3 or 4 times per week	3 (5.5)	30 (22.4)	
5 or 6 times per week	5 (9.2)	19 (14.2)	
Once a day	2 (3.7)	39 (29.1)	
2 or 3 times per day	1 (1.8)	9 (6.7)	
4 or 5 times per day	0 (0)	1 (0.7)	
6 or more times per day	0 (0)	0 (0)	
			**<0.001**
Legumes			
Never or almost never	8 (14.8)	5 (3.7)	
1 to 3 times per month	8 (14.8)	17 (12.7)	
1 or 2 times per week	35 (64.8)	78 (58.2)	
3 or 4 times per week	3 (5.5)	30 (22.4)	
5 or 6 times per week	0 (0)	2 (1.5)	
Once a day	0 (0)	2 (1.5)	
2 or 3 times per day	0 (0)	0 (0)	
4 or 5 times per day	0 (0)	0 (0)	
6 or more times per day	0 (0)	0 (0)	
			**0.01**
Olive oil			
Never or almost never	3 (5.5)	0 (0)	
1 to 3 times per month	5 (9.2)	2 (1.5)	
1 or 2 times per week	7 (12.9)	7 (5.2)	
3 or 4 times per week	12 (22.2)	29 (21.6)	
5 or 6 times per week	21 (38.9)	78 (58.2)	
Once a day	6 (11.1)	16 (11.9)	
2 or 3 times per day	0 (0)	2 (1.5)	
4 or 5 times per day	0 (0)	0 (0)	
6 or more times per day	0 (0)	0 (0)	
			**<0.001**
Refined grains			
Never or almost never	5 (9.2)	29 (21.6)	
1 to 3 times per month	3 (5.5)	7 (5.2)	
1 or 2 times per week	11 (20.4)	30 (22.4)	
3 or 4 times per week	8 (14.8)	20 (14.9)	
5 or 6 times per week	5 (9.2)	6 (4.5)	
Once a day	14 (25.9)	16 (11.9)	
2 or 3 times per day	6 (11.1)	24 (17.9)	
4 or 5 times per day	2 (3.7)	0 (0)	
6 or more times per day	0 (0)	2 (1.5)	
	0 (0)	0 (0)	**0.03**

Whole grains			
Never or almost never	18 (33.3)	19 (14.2)	
1 to 3 times per month	5 (9.2)	7 (5.2)	
1 or 2 times per week	13 (24.1)	31 (23.1)	
3 or 4 times per week	7 (12.9)	28 (20.9)	
5 or 6 times per week	1 (1.8)	12 (8.9)	
Once a day	10 (18.5)	29 (21.6)	
2 or 3 times per day	0 (0)	8 (6.0)	
4 or 5 times per day	0 (0)	0 (0)	
6 or more times per day	0 (0)	0 (0)	
			**0.01**
Other oils			
Never or almost never	18 (33.3)	55 (41.0)	
1 to 3 times per month	13 (24.1)	44 (32.8)	
1 or 2 times per week	6 (11.1)	24 (17.9)	
3 or 4 times per week	5 (9.2)	5 (3.7)	
5 or 6 times per week	0 (0)	2 (1.5)	
Once a day	12 (22.2)	4 (3.0)	
2 or 3 times per day	0 (0)	0 (0)	
4 or 5 times per day	0 (0)	0 (0)	
6 or more times per day	0 (0)	0 (0)	
		0 (0)	**<0.001**
Eggs			
Never or almost never	0 (0)	4 (3.0)	
1 to 3 times per month	2 (3.7)	5 (3.7)	
1 or 2 times per week	27 (50.0)	54 (40.3)	
3 or 4 times per week	18 (33.3)	44 (32.8)	
5 or 6 times per week	5 (9.2)	13 (9.7)	
Once a day	2 (3.7)	11 (8.2)	
2 or 3 times per day	0 (0)	3 (2.2)	
4 or 5 times per day	0 (0)	0 (0)	
6 or more times per day	0 (0)	0 (0)	
	0 (0)	0 (0)	
			0.56
Pastry			
Never or almost never	17 (31.5)	79 (59.0)	
1 to 3 times per month	13 (24.1)	26 (19.4)	
1 or 2 times per week	9 (16.7)	15 (11.2)	
3 or 4 times per week	2 (3.7)	7 (5.2)	
5 or 6 times per week	4 (7.4)	1 (0.7)	
Once a day	5 (9.2)	6 (4.5)	
2 or 3 times per day	3 (5.5)	0 (0)	
4 or 5 times per day	1 (1.8)	0 (0)	
6 or more times per day	0 (0)	0 (0)	
			**<0.001**

Sugar			
Never or almost never	15 (27.8)	58 (43.3)	
1 to 3 times per month	8 (14.8)	20 (14.9)	
1 or 2 times per week	3 (5.5)	13 (9.7)	
3 or 4 times per week	0 (0)	10 (7.4)	
5 or 6 times per week	0 (0)	4 (3.0)	
Once a day	14 (25.9)	23 (17.1)	
2 or 3 times per day	11 (20.4)	5 (3.7)	
4 or 5 times per day	1 (1.8)	1 (0.7)	
6 or more times per day	2 (3.7)	0 (0)	
			**<0.001**
Alcohol			
Never or almost never			
1 to 3 times per month	32 (59.2)	63 (47.0)	
1 or 2 times per week	10 (18.5)	26 (19.4)	
3 or 4 times per week	6 (11.1)	29 (21.6)	
5 or 6 times per week	3 (5.5)	7 (5.2)	
Once a day	2 (3.7)	0 (0)	
2 or 3 times per day	1 (1.8)	6 (4.5)	
4 or 5 times per day	0 (0)	1 (0.7)	
6 or more times per day	0 (0)	2 (1.5)	
	0 (0)	0 (0)	
			0.17
Water			
Never or almost never	2 (3.7)	3 (2.2)	
1 to 3 times per month	0 (0)	0 (0)	
1 or 2 times per week	0 (0)	0 (0)	
3 or 4 times per week	0 (0)	3 (2.2)	
5 or 6 times per week	0 (0)	0 (0)	
Once a day	2 (3.7)	6 (4.5)	
2 or 3 times per day	11 (20.4)	15 (11.2)	
4 or 5 times per day	17 (31.5)	24 (17.9)	
6 or more times per day	22 (44.7)	83 (61.9)	
			0.06

Supplementary Table S3.- Questions and criteria to calculate the 14-MEDAS score.

**Questions from FFQ-MEDAS**	**Response options and scoring criteria to calculate MEDAS from FFQ-MEDAS**
1.Do you use olive oil as your main culinary fat?	Yes = 1<br>No = 0
2. How many tablespoons of olive oil do you consume per day?	Four or more = 1<br>One or less; two or three = 0
3. How many vegetable servings do you consume per day? (Including cooked and raw vegetables; potatoes and beans are not included; one serving = one large cup or half a large plate)	Two; three or more = 1<br>Less than one; one; two = 0
4. How many servings of fresh fruit do you consume per day? (One serving = one fruit unit of medium size, one large cup of sliced fruit, one slice of melon or watermelon of medium size, or one cup of freshly squeezed juice).	Three or more = 1<br>Less than one; one; two = 0
5. How many servings of red meat, or red meat products do you consume per week? (Red meat: veal/beef, pork, lamb; derived products: hamburguers, sausages, ham, etc, you may include here some examples of meat products typical from your country. One serving = 100 to 150 g = a quarter to half a meal dish)	One or less; two to four; five to six = 1<br>Seven or more =0 (corresponds to one or more per day)
6. How many servings of butter, margarine, or cream do you consume per day? (One serving = 12 g = one dessert spoon of butter and margarine; 2 tablespoons for cream.)	Less than one = 1<br>One; more than one = 0
7. How many sweet/fizzy beverages/sodas do you consume per day? (You may include some examples of juice fruits or soft drinks with added sugar commonly consumed in the country)	Less than one = 1<br>One; more than one = 0
8. How many glasses/cups of wine do you consume per week?	Seven to fourteen glasses (one or two glasses per day) =1<br>one or less (occasionally); two to six (sometimes but not daily); more than fourteen (more than two glasses per day) = 0
9. How many servings of legumes do you consume per week? (Including beans, peas, chickpeas, lentils, etc. One serving = 150 g = 1 plate or 1 cup.)	Three or more = 1<br>Less than one; one; two = 0
10. How many servings of fish or shellfish do you consume per week? (One serving = 100 to 150 g = a quarter to half a meal dish)	Three or more = 1<br>Less than one; one; two = 0
11. How many times per week do you consume industrial (not homemade) desserts/sweets/pastries? (Including cakes, cookies, biscuits, ice-creams, custard, etc)	Less than one; one; two = 1<br>Three; four or more = 0
12. How many servings of (unsalted) nuts do you consume per week? (Including unsalted peanuts, almonds, hazelnuts, chestnut, walnuts, pecan nuts, etc. One serving = 30 g = one handful)	Three or more = 1<br>Less than one; one; two = 0

13. Do you preferentially consume chicken, turkey or rabbit meat, or a vegetarian protein source, instead of red meat or any derived products?	Yes = 1 (1 point for vegetarians)<br>No = 0
14. How many times per week do you consume dishes cooked with tomato or tomato sauce, onion and (or) garlic, and olive oil?	Two or more = 1<br>Less than one; one = 0
